# Chronicles of Nanoerythrosomes: An Erythrocyte-Based Biomimetic Smart Drug Delivery System as a Therapeutic and Diagnostic Tool in Cancer Therapy

**DOI:** 10.3390/pharmaceutics13030368

**Published:** 2021-03-10

**Authors:** Shamama Javed, Sultan Alshehri, Ambreen Shoaib, Waquar Ahsan, Muhammad Hadi Sultan, Saad Saeed Alqahtani, Mohsin Kazi, Faiyaz Shakeel

**Affiliations:** 1 Department of Pharmaceutics, College of Pharmacy, Jazan University, P.O. Box 114, Jazan 45142, Saudi Arabia; sjahmad@jazanu.edu.sa (S.J.); mhsultan@jazanu.edu.sa (M.H.S.); 2 Department of Pharmaceutics, College of Pharmacy, King Saud University, P.O. Box 2457, Riyadh 11451, Saudi Arabia; salshehri1@ksu.edu.sa (S.A.); mkazi@ksu.edu.sa (M.K.); 3 Department of Pharmaceutical Sciences, College of Pharmacy, Almaarefa University, Riyadh 11597, Saudi Arabia; 4 Department of Clinical Pharmacy, College of Pharmacy, Jazan University, P.O. Box 114, Jazan 45142, Saudi Arabia; asahmad@jazanu.edu.sa (A.S.); ssalqahtani@jazanu.edu.sa (S.S.A.); 5 Department of Pharmaceutical Chemistry, College of Pharmacy, Jazan University, P.O. Box 114, Jazan 45142, Saudi Arabia; wmohammad@jazanu.edu.sa

**Keywords:** nanoerythrosome, nanoerythrocyte, nanovesicles, biomimetic, cancer therapy, diagnostics, imaging agents

## Abstract

Recently, drug delivery using natural biological carriers has emerged as one of the most widely investigated topics of research. Erythrocytes, or red blood cells, can act as potential carriers for a wide variety of drugs, including anticancer, antibacterial, antiviral, and anti-inflammatory, along with various proteins, peptides, enzymes, and other macromolecules. The red blood cell-based nanocarrier systems, also called nanoerythrosomes, are nanovesicles poised with extraordinary features such as long blood circulation times, the ability to escape immune system, the ability to release the drug gradually, the protection of drugs from various endogenous factors, targeted and specified delivery of drugs, as well as possessing both therapeutic and diagnostic applications in various fields of biomedical sciences. Their journey over the last two decades is escalating with fast pace, ranging from in vivo to preclinical and clinical studies by encapsulating a number of drugs into these carriers. Being biomimetic nanoparticles, they have enhanced the stability profile of drugs and their excellent site-specific targeting ability makes them potential carrier systems in the diagnosis and therapy of wide variety of tumors including gliomas, lung cancers, breast cancers, colon cancers, gastric cancers, and other solid tumors. This review focuses on the most recent advancements in the field of nanoerythrosomes, as an excellent and promising nanoplatform for the novel drug delivery of various drugs particularly antineoplastic drugs along with their potential as a promising diagnostic tool for the identification of different tumors.

## 1. Introduction

Erythrocytes (ER) or the red blood cells (RBC) are the most abundant blood cells responsible for the transport of gases from lungs to the tissues [1]. Over the past two decades, they have been exploited to develop various biomimetic cell-based carrier systems, including resealed erythrocytes (RE), genetically engineered RBCs, as well as RBC-membrane coated nanoparticles (NP) (RBC-m-NP) for various biomedical applications. Their transition from preclinical studies to clinical stage is a proof of being smart and effective enough to be used as novel drug delivery vehicles. Other than erythrocytes, other blood cells such as platelets and leukocytes can also be used as carrier system for a variety of drugs [2]. All these cell-based drug carrier systems are superior and have various advantages over other drug-delivery systems such as micro- and nano-particles in terms of properties such as prolonged circulation time, biocompatibility, biodegradability, non-immunogenicity, ease of preparation, and flexibility in use [3], apart from improved patient compliance by extending the time of release of drugs from these systems. Escaping the rapid clearance from body and specificity for the target are other major advantages associated with such carriers [4]. These extraordinary features helped the carrier erythrocytes (C-ER) to emerge as potential drug-carrier systems for the therapy of cellular infections, cardiovascular disorders, cancer therapy, gene therapy, and other ailments [5]. Recently, several other cell-based drug delivery strategies such as bacterial ghosts, dendritic cells (DC) and genetically engineered stem cells have also emerged owing to the successful application of C-ERs [6].

Nanoerythrosomes (NER) are the C-ERs which are recognized as modern day, novel, and smart drug delivery systems associated with increased bioavailability, improved pharmacokinetics, and low toxicity [7]. As a physiological carrier, NERs can release the drug in circulation for weeks, have high loading capacity, are easy to be processed and have good ability to accommodate biologics, antigens, contrasting agents, peptides, proteins, enzymes, and macromolecules using different chemical and physical based methods [8,9]. Innumerable examples are available in the literature discussing the drug delivery by C-ERs for applications in drug targeting to the reticuloendotehlial system (RES), enzyme therapy and improvement in the delivery of oxygen to the tissues [10,11]. One of such example is the delivery of bovine serum albumin (BSA) as a model antigen, which can be loaded on the human erythrocytes and delivered to the RES [12]. C-ERs are the unique drug delivery system that is also known to be capable of modulating the immune responses [13]. Amongst different antineoplastic drugs, methotrexate, etoposide, doxorubicin, and carboplatin have been successfully encapsulated in these carriers previously [14]. C-ERs are shown to protect the carried therapeutics such as antibiotics, antineoplastics, corticosteroids, peptides, and enzymes from being inactivated by different endogenous factors. The three main mechanisms, by which these C-ERs work are prolongation of circulation half-life, slow drug release, and organ-specific targeting [15]. Regulatory aspects of carrier erythrocytes and industrial developments are evolving day by day. However, their production scalability, process validation and quality control is still challenging enough for successful industrialization [16].

This review focuses on the NERs which have emerged as a promising and versatile platform for the successful delivery of a number of drugs in particular the antineoplastic drugs. Recent advancements in the field of NERs along with the nanoformulations developed and used in the cancer and non-cancer therapy are covered. The diagnostic and imaging applications of NERs, as well as the recent patents and clinical trials associated with these carriers have also been discussed.

## 2. Fabrication of NERs

Various methods have been successfully utilized for the fabrication of NERs in order to enhance membrane permeability and cellular targeting of therapeutic agents. These methods are described below [17,18,19,20].

### 2.1. Dilutional Hemolysis and Resealing Method

Hemolysis and resealing method is commonly used method for drug loading into erythrocytes. In this technique, the erythrocytes are exposed to hypotonic solution (0.4% *w/v* sodium chloride) until it reaches a critical value of volume or pressure in order to rupture the cells. Then, resealing is carried out in order to restore osmotic properties [19]. Dilutional hemolysis utilizing hypotonic solution (0.4% *w/v* sodium hydroxide) at 0 °C and resealing at 37 °C has been utilized as cellular carriers for the successful delivery of low molecular weight drugs like β-glucosidase and β-galactosidase [20]. This technique is the simplest and fastest technique for the preparation of NERs. The main disadvantages of this technique are low drug loading efficiency (1–8%) and considerable loss of hemoglobin and other cellular components [19,20].

### 2.2. Preswell Dilutional Hemolysis Method

The preswell dilutional hemolysis technique has been developed for the improvement of drug loading efficiencies. In this technique, an initial swelling of erythrocytes is achieved using slightly hypotonic solution, followed by low-speed centrifugation. The drug loading efficiencies of some drugs such as ibuprofen and thyroxin have been significantly increased using preswell dilutional hemolysis technique compared to dilutional hemolysis technique [17].

### 2.3. Hypotonic Dialysis Method

The main problems of dilution techniques is the low drug-loading efficiencies. This problem of such dilutional techniques can be overcome using hypotonic dialysis technique. In hypotonic dialysis technique, the lysis and resealing are carried out within dialysis tube. This technique offers high drug entrapment efficiency, high drug-loading efficiency, and high cells recovery compared to dilutional techniques. However, long processing time and special instruments are required for this technique. This method has been successfully utilized for the loading of various enzymes (asparginase, galactosidase, and glucoserebrosidase, etc.) and drugs (gentamicin, adriamycin, and furamycin, etc.) [17,21].

### 2.4. Use of Red Cell Loader

The instrument like red cell loader is used for the loading of non-diffusible drugs into erythrocytes. Using this technique, the drugs are loaded into small volume of blood within 2 h at room temperature under blood banking conditions. The drug loading up to 30% and cell recovery of 30–50% can be achieved using this technique [18].

### 2.5. Isotonic Osmotic Lysis

It is also known as osmotic pulse technique which can be achieved by chemical or physical means. The chemical which are used to achieve isotonic hemolysis are urea, polyethylene glycols, and ammonium chloride. This technique is not immune to the changes in the composition of membrane structures [17,18].

### 2.6. Membrane Perturbation Technique

This technique is based on the enhancement in the membrane permeability of erythrocytes when the cells are exposed under certain chemicals or drugs. The membrane permeability of erythrocytes was enhanced under exposure to an antibiotic amphotericin B. This technique has been applied to load an antitumor drug daunomycin into erythrocytes of rat and human. However, this technique is irreversible and hence not very popular [17,18].

### 2.7. Lipid Fusion Technique

This technique is used for the loading of drug-loaded lipid vesicles into erythrocytes. Drug-loaded lipid vesicles can be directly fused into human erythrocytes, which could result into exchange with a lipid-entrapped drug. This technique has been successfully applied for the loading of inositol monophosphate into human erythrocytes for the improvement of oxygen carrying capacity of the cells. However, the drug-loading capacity of this technique is too low (1%) [18,19].

Some other techniques such as extrusion, sonication, and electrical breakdown techniques have also been utilized for the loading of drugs into human erythrocytes [18].

## 3. NERs as an Efficient Drug Delivery Tool

**Figure 1:**
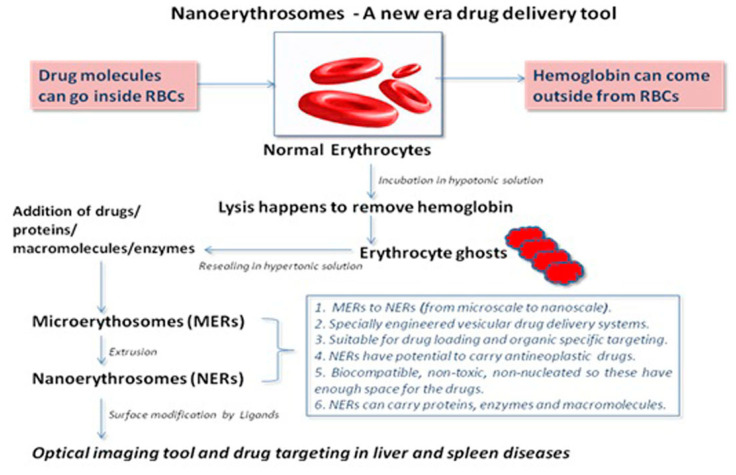
Preparation of nanoerythrocytes (NER) from normal erythrocytes (ER).

The compendium of knowledge and several research studies available on the RBC-based carrier systems led to the development of more advanced RBC-membrane derived liposomes also called as NERs. It has opened a new vista of its futuristic application in the field of nanomedicine with greater potentials. These are NPs often designated with other names such as functionalized-NPs, hybrid-NPs, biomimetic-NPs, and targeted-NPs in the literature [22,23,24]. The NERs, being nanosized derivatives of erythrocytes, exhibit important properties such as biodegradability, biocompatibility, excellent release profiles, and organ and site-specific targetability. The prolonged circulation time in blood for NERs is owing to the higher surface area-to-volume ratio than the parent erythrocytes (approximately 80 times higher). Extra care is necessary while handling parent erythrocytes for maximum drug loading. The hemoglobin and cytoplasmic contents of the erythrocytes are removed using a controlled hypertonic solution mediated hemolysis followed by the loading of therapeutic agents. This process of removal of cellular contents should be in such a controlled manner that the plasma membrane should not be disrupted and the cells are allowed to form seamless nanosized capsule. One erythrocyte can be fragmented into thousands of NERs depending upon the recovered cell percentage post removal of its organelles. The NERs are prepared from the hemoglobin (Hb)-free erythrocyte ghosts using sonication, extrusion, and electrical impulses techniques. Uniform nanosized NERs are obtained using extrusion technique which is the most efficient technique amongst all. The nanosizing technique, lysis media, and processing temperature have considerable impact on the development of stable and viable NERs-based formulations. Owing to the biomimetic properties of NERs, these can be utilized for the efficient encapsulation of peptides, enzymes, genetic materials, toxins, and contrasting agents which can be applied for the treatment of liver, lymph nodes, and spleen disorders, as well as various types of carcinomas [23,24,25]. Similar to liposomes, NERs are the nanovesicles prepared by the extrusion of RBC ghosts, are membrane-covered nanoscale containers, discrete spherical in shape, with unilamellar membrane of 4.5 µm thickness and having diameter up to 100 nm (Figure 1). However, the disadvantages associated with liposomes and monoclonal antibodies are not present in NERs and these show excellent colloidal stability in both buffer and serum at room temperature [25].

## 4. Applications of NERs in Cancer Therapy and Diagnosis

### 4.1. NERs in Cancer Therapy

The important studies on NERs and their formulations for the application in cancer therapy are summarized in Table 1. NERs are artificial, synthetic, biomimetic, and presently the most investigated and sought after RBC cell-based carrier systems under this category. These RBC cell-based carrier systems have covered a path of progress from conventional REs (microscale) to the most advanced NERs (nanoscale). These RBC-nanovesicles (RBC-NV) are fused onto the core NPs through extrusion, sonication, and electroporation techniques. Being biomimetic NPs, NERs attain specific functions including ligand recognition, tumor-targeting, longer blood circulation time, and ability to escape the immune system. These activities lead to the core-shell interactions, mimicking the source cells, and improving the therapeutic efficacy of drugs through specific delivery and enhanced drug-accumulation inside the tumor [26]. NERs marked the beginning of an unparalleled approach towards the therapy of a number of diseases including cancer owing to multiple advantages such as small size, ability to encapsulate antineoplastic drugs, and organ-specific targeting which further gave them added advantages over their other carrier counterparts in cancer nanomedicine. The surface of NERs can be modified using tumor-targeting ligands, which further improve the drug efficacy and optimize drug pharmacokinetics.

**Figure 2:**
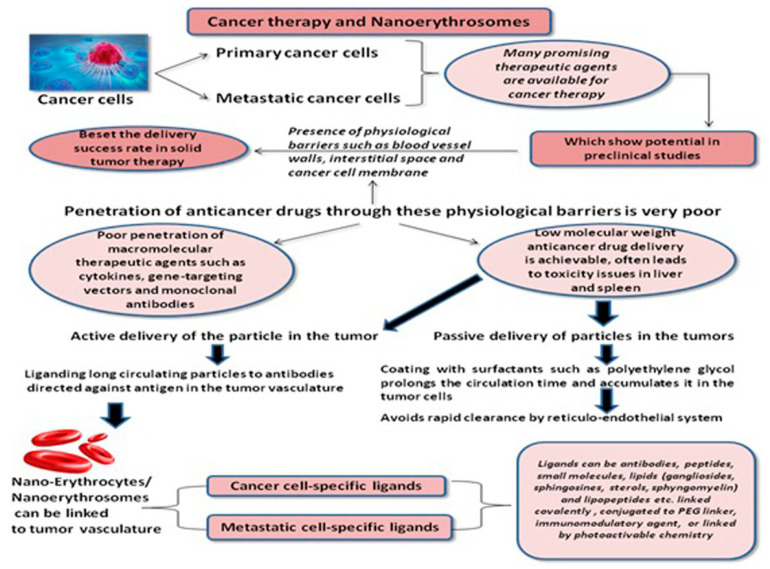
Role of NERs in cancer therapy.

The loading and delivery of antineoplastic drugs using various pharmaceutical and nanotechnological engineering techniques is one of the most important applications of NERs. The unique techniques of surface modification and functionalization provide additional attractive feature to this delivery system and further increase its potential in cancer therapy (Figure 2).

Numerous studies have been performed in the field of cancer therapy using NERs over the last two decades and multitude of its medical applications in other ailments diversify their applications in the arena of drug delivery as therapeutic, diagnostic, theragnostics, and imaging tool [27]. The nanomedicines have the ability to deliver the drugs directly to the individual organs to avoid the adverse or side effects; however, the drug delivery by conventional nanocarriers is limited by difficulty in delivering to most target organs and dire need of the affinity moieties [28].

The poor drug loading, low anti-tumor efficacy, and rapid clearance from the blood circulation are the major challenges faced by the conventional anti-cancer nanocarriers. The most challenging part is the high uptake of nanocarriers by the liver and spleen because of RES. The majority of nanocarriers injected intravascularly end up in the liver causing hepatotoxicity [29]. The NERs, as an innovative drug delivery system, provides with high drug loading capacity, specificity in targeting tumor cells, and synergistic anti-cancer sensitization using human erythrocyte membrane as delivery platform. This novel technology can be used in treating and preventing cancer metastasis such as breast cancer metastasis, mainly by blocking the circulation of metastatic cancer cells by inhibiting angiogenesis and capturing circulating endothelial progenitors that are recruited to the tumor. They also physically block (infarction) the capillaries of the tumor or the metastasis. The inhibition of molecular pathways of the metastatic invasion and cancer growth is the latest therapeutic approach for cancer metastasis. The physical blocking of metastasis and angiogenesis by incorporating tumor-and angiogenesis-specific ligands such as antibodies, small molecules and peptides into the plasma membrane of erythrocytes is very novel [30].

**Figure 3:**
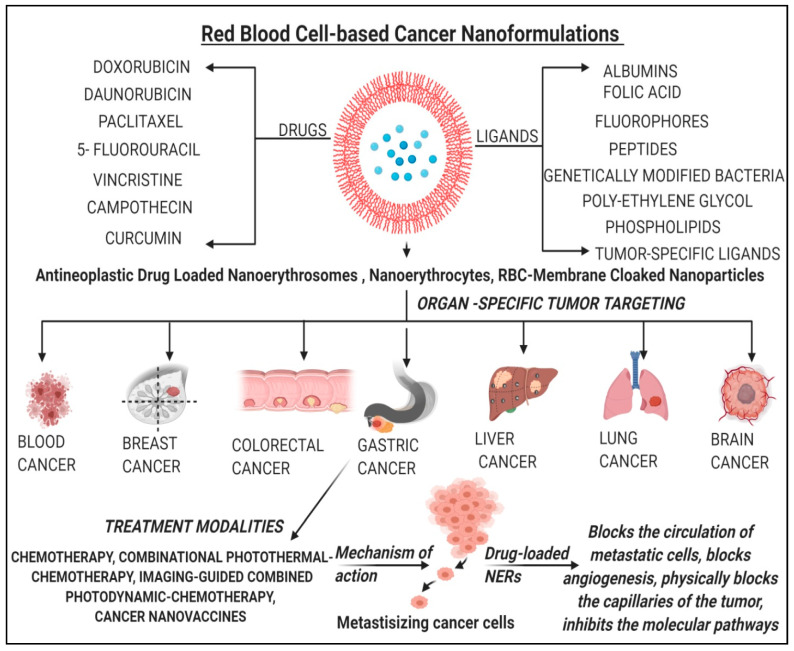
Summary of red blood cell (RBC)-based anticancer nanoformulations (anti-cancer-loaded-NERs) along with their mechanisms.

Polymeric-NPs as intravascular drug delivery vehicles have been studied extensively in past but their applications are beset by rapid clearance from the circulation by RES. This led to the development of NERs as novel cancer drug delivery systems for the optimal delivery of various anticancer pharmaceutical agents. These anticancer drugs, when administered intravenously, cannot reach the tumor site efficiently owing to their weal specificity which results into lower therapeutic potential and serious side effects. One of the major reasons of multidrug resistance (MDR) and thereby chemotherapy failure is the efflux of anticancer drugs mediated by P-glycoprotein (P-gp). This problem is overcome to an extent when these anticancer agents are delivered to the tumors in combination with P-gp inhibitors and this has proven to be an effective strategy [31]. An organ-specific or site-specific nano-drug delivery system based on human RBCs is a newer and effective strategy of the present decade (Figure 3).

The anticancer drug delivery using NERs was first carried out by a team of researchers two decades ago in 1994, when they explored the anticancer drug daunorubicin (DAU) and prepared the DAU-NERs for enhanced antineoplastic potential. DAU was linked covalently to the NERs using glutaraldehyde as homobifunctional linking arm, and it was observed that the cytotoxicity of conjugated drug was higher as compared to the free drug when assessed on P388D1 cancer cell line [32]. Results indicated that these DAU-NER conjugates could potentially be utilized as drug carriers in CDF1 leukemia tumors. The mechanism behind the cellular uptake was neither the diffusion through cell membrane nor endocytosis but free DAU was found to slowly release by hydrolysis of glutaraldehyde linking arm, producing a high concentration of free DAU in the cells vicinity for a long period of time [33], and phagocytosis of DAU-NER complex was not involved [34]. The biodistribution of 125I-NERs purified by dialysis followed by intravenous (IV) and intraperitonial (IP) injections in CD1 mice was investigated in another study. NERs were rapidly (less than 30 min) removed from the systemic circulation after IV administration, however, the IP administration showed a higher uptake by liver and spleen, accumulation in lungs and marked activity was observed in the inguinal lymph nodes after 2 h of the administration [35].

Earlier, the surface modification of NPs could be achieved using polymers such as poloxamines, poloxamers and polyethylene glycols, to avoid opsonization, but now, these NPs could be anchored to the surface of RBCs to escape the clearance by RES as the RBCs are able to do so. This leads to a novel approach in the intravascular drug delivery system as RBC-anchored NPs. The RBC-anchored polymeric NPs were prepared using polystyrene NPs modified with carboxyl groups (110, 220, 450, and 1100 nm in diameter). An improved in vivo circulation lifetime was achieved using RBC-anchored NPs [36]. In another study by the same research group, RBC-anchored NPs were eventually get detached from RBCs owing to the shear forces and subsequently get cleared up in the liver and spleen [37]. The surface modifications of NERs is possible with glutaraldehyde, antibodies, and carbohydrates (sialic acid), which improve their target specificity and prolong their in vivo circulation time, helps escape the RES and protects the drug from the inactivation by endogenous chemicals and enzymes [38,39]. Based on this approach, a top-down biomimetic approach was reported utilizing particle functionalization. The fluorophore-loaded biomimetic NPs were prepared using poly (*d,l*-lactide-co-glycolide) (PLGA) polymer and fused with RBC-derived vesicles. The prepared RBC-fused biomimetic NPs were injected in mice. Results revealed superior circulation half-life in cases of biomimetic-NPs as compared to control group-NPs which were coated with conventional synthetic stealth materials. An increased particle retention time of 72 h in the blood was observed which proved the approach as very unique and effective in the NPs functionalization [40]. In another study, RBC-membrane-cloaked polymeric NPs were prepared by lipid-insertion technique utilizing fluorescein isothiocyante (FITC)-polyethylene glycol (PEG)-lipid. The FITC-PEG-lipid NPs were functionalized with folate and nucleolin-targeting aptamer AS1411 showed a receptor-specific targeted delivery when tested against the cancer cell lines models [41]. Another promising integration of cell-based drug delivery and nanotechnology was seen when RBCs were linked to iron oxide-NPs pre-coated with chlorine-e^6^ and a well-known chemotherapeutic drug, doxorubicin (DOX) was loaded for an imaging-guided combined chemotherapy-photodynamic therapy of carcinomas. Interestingly, the marked synergism and excellent therapeutic results were observed in animal tumor model [42]. In another report, the oxygen self-enriched biomimetic RBCs were prepared by cloaking the albumin-NPs containing indocyanine green (ICG) and perfluorocarbon (PFC) with RBC membranes. The rapid clearance of conventional nanocarriers by RES and low singlet oxygen quantum (0.08) of ICG often resulted into limited phytotherpaeutic efficacy. The prepared oxygen self-enriched RBCs had long circulation time, high oxygen carrying capacity, and biomimetic properties ideal for clinical cancer phototherapy treatment [43]. Another novel NERs-ghost delivery system was developed to transport sodium transhinone-IIA sulphonate (STS-Nano-RBC) for IV use in rats. These nanosystems had narrow particle size distribution, good stability for 21 days, and better loading efficiency as compared to conventional STS injections in vivo [44].

**Figure 4:**
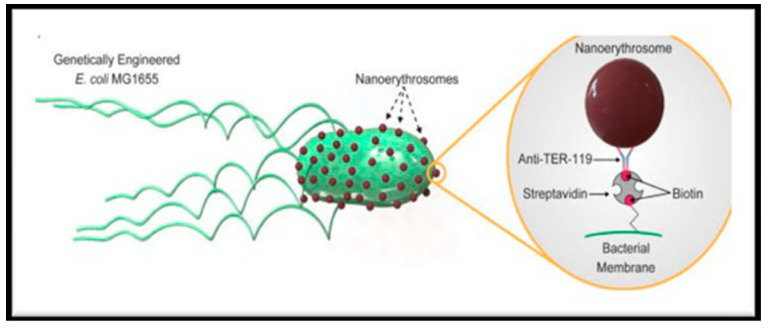
Biohybrid microswimmers using genetically modified *E. coli* bacterium and NERs for targeted cargo-delivery (Image adopted from reference [50], APL Bioeng, 2020, which was published under a creative commons attribution (CC BY) license).

BackgroundIt is now evident that the adverse effects associated with the conventional cancer therapy (chemotherapy, surgery, and radiotherapy) can effectively be addressed by applying the nanomedicine approach. Nano-engineering makes targets achievable and with diverse surface functionalization methods, nowadays RBCs are not only targeting drug carriers but therapeutic and imaging diagnostic tools as well, what we commonly term as theranostics [45]. Recently, RBC membrane-loaded with hydrophobic drug camptothecin were prepared which were non-covalently labeled with amphiphilic fluorophore nanovesicles (NVs). These NVs were overall stable, nonphagocytic and exhibited minor stimulation of macrophages for cytokine release. Intravenously injected NVs in balb/c mice exhibited better retention over 48 h and minimal accumulation in vital organs such as heart, liver, and kidneys [46]. NE-membrane-chaperoned 5-fluorouracil (5-FU) liposomes were also prepared as biomimetic delivery system to target hepatocellular carcinoma cell lines. Liposomes of 5-FU were prepared by thin film hydration method using dipalmitoyl phosphatidylcholine (DPPC) and cholesterol. NE-membrane-chaperoned 5-FU liposomes were prepared by extrusion technique. The drug-targeting capability of NE-membrane-chaperoned liposomes was higher than conventional 5-FU solution [47]. The most recent advances in this field include antigen delivery systems using NERs in which the tumor antigens are loaded onto the NERs using fusion tumor cell membranes. This nanosystem was shown to markedly inhibit the tumor growth in B16F10 and 4T1 tumor models in vivo [48]. A new NER-tailoring technique evolved recently, wherein, the NERs in the size range of 200 to 300 nm were produced by addition of phospholipids DPPC to ghost RBCs [49]. In yet other significant research, bio-hybrid microswimmers were developed which had the integration of motile-microorganisms with artificial cargo carriers to revolutionize targeted-cargo delivery. In this study, genetically-engineered flagellated *Escherichia coli* species were integrated with RBC-derived NERs through conjugation of streptavidin-modified microorganisms with biotin-modified NERs using non-covalent streptavidin-biotin interactions. The high fabrication efficiency and good motility performance was achieved with these personalized biohybrid microswimmers, as shown in Figure 4 [50].

### 4.2. NERs in Immunotherapy

The main focus of cancer immunotherapy is to manipulate the patient’s own immune system to recognize and destroy the cancer cells. The NPs-based drug delivery systems show their therapeutic potential by evading the immune system of hosts and delivering the drug efficiently and safely to the site of action. In lieu of this, an erythrocyte membrane-enveloped PLGA NPs (PLGA-NP) based nanovaccine was developed for hgp 10025-33, an antigenic peptide and monophosphoryl lipid (MPLA), a toll-like receptor-4 agonist. To target the antigen presenting cells (APC), a mannose-inserted membrane structure was constructed in the lymphatic organ and the prepared nanovaccine showed increased retention of protein content in the erythrocytes, improved in vitro cellular uptake, inhibition of tumor growth, prolonged tumor-occurring time, and reduced tumor metastasis, resulting in a potential nanoplatform for the delivery of antigens in the immunotherapy of cancer [51]. After the conventional mono-chemotherapy, research was carried out on combination chemotherapy approach for cancer treatment by these carrier systems. A programmed co-delivery of paclitaxel (PAX) and DOX as the hydrophilic-lipophilic chemotherapeutic drug pair into the magnetic *O*-carboxymethyl-chitosan NPs further masked using Arg-Gly-Asp anchored erythrocyte membrane was achieved and compared with the conventionally prepared PEG coated NPs. Superior circulation time, improved tumor accumulation, and better tumor uptake was demonstrated using biomimetic-NPs with much lower side effects as compared to their counterpart [52].

In an attempt to counter the insufficient targeting ability of NERs which beset their clinical applications [53], several PAX-loaded polymeric NPs were linked to the biomimetic 4T1-breast cancer cell membranes. Polymeric NPs were prepared using poly(caprolactone) and pluronic copolymer F68. The novel nanoformulation demonstrated superior interactions with the source tumor cells, prolonged circulation time, and higher cell-specific targeting of the homotypic primary tumors and metastasis [54]. The in-combination advantages of erythrocytes and NPs have so far offered three types of RBC-based nanomedicines: (i) whole erythrocytes, (ii) cell membrane-coated NPs, and (iii) NERs and together they offer optimal blend of important features as a multifunctional platform in the cancer immunotherapy [55]. Similarly, curcumin-loaded NERs (CUR-NER) were prepared by sonication method showing nearly uniform spheres with diameter (245.7 ± 1.3 nm), encapsulation efficiency (50.65% ± 1.36%) and loading efficiency (6.27% ± 0.29%). These CUR-NERs were efficiently taken up by the cancer cells which displayed good anti-tumor efficacy [56].

The molecular targeting of the RBC membrane glycophorin-A (GPA) receptor can mediate the membrane attachment of protein based-therapeutics which can open diverse applications of NERs for preclinical and clinical biocompatibility [57]. A cell membrane-coated NP platform for the new antitumor drug gambogic acid (GA) was developed in order to improve its limited clinical applications owing to its poor aqueous solubility. The biomimetic nanocarrier system was prepared using PLGA polymer. The prepared PLGA nanocarrier system was found to show improved stability and biocompatibility. In addition, the GA-loaded RBC-m- PLGA-NPs showed better antitumor efficacy and lesser toxicity as compared with the free GA in the treatment of colorectal cancer [58]. In another study, a novel RBC-based artificial APC (aAPC) system was developed which can mimic the antigen presenting dendritic cells (DC) and activate T-cells. This system had promising applications in the transfer of adoptive T-cells and even in the activation of circulating T-cells directly for the immunotherapy of cancer [59].

### 4.3. NERs in Cancer Imaging and Diagnostics

For the in vivo cancer imaging, RBC-up-conversion NPs (RBC-UCNP) were developed for cancer targeting and imaging recently. UCNPs were functionalized using 1,2-distearoyl-sn-glycero-3-phosphoethanolamine-*N*-[methoxy(polyethylene glycol)-2000] (ammonium salt) (DSPE-PEG). RBC-UCNPs were obtained using extrusion method and showed superior chemical and optical features. These RBC-UCNPs were utilized successfully for in vivo tumor imaging [60]. The biomimetic RBC membrane-coated ferric oxide (Fe_3_O_4_) magnetic NPs (RBC-MNP) prepared using microfluidic electroporation strategy also exhibited better treatment effect in imaging-guided cancer therapy [61]. Therefore, biomimetic-NPs derived from erythrocytes, white blood cells, cancer cells, stem cells, platelets, or bacterial ghost cells are engineered therapeutic carrier system presently garnering lot of attention for their applications in diagnostics, imaging, drug delivery, vaccines, and immune-modulation, etc. [62]. The limitations of fluorescence image-guided tumor surgery and photodynamic therapy including lesser penetration depth, lower signal-to-noise ratio of traditional first near infra-red (NIR) window (NIR-I) fluorescence, as well as the hypoxic tumor environment can be overcome by RBCs-based multimodal probe. This probe was able to show improved tumor targeting and fluorescent probes retention post IV injection, therefore, the second NIR window (NIR-II) fluorescence bioimaging-guided absolute tumor resection and highly efficient photodynamic therapy could be achieved [63].

### 4.4. NERs in Cancer Combination Therapy

In continuation to explore the cancer combination therapy using NERs, a hybrid biomimetic RBCs-melanoma cells (B16-F10) coating (RBC-B16 hybrid membrane) along with the loading of camouflaged DOX on the hollow copper sulfide (CuS)-NPs were developed for the melanoma therapy and compared with the bare CuS-NPs. Interestingly, the DOX-loaded RBC-B16-coated CuS-NPs exhibited excellent synergistic effects as the photothermal-chemotherapeutic agent and were 100% effective in inhibiting the melanoma tumor growth [64]. Small molecular drug co-assemblies of 10-hydroxycampthothecin (10-HCPT) and ICG were fabricated in RBC membranes as a biomimetic and combinational therapeutic tool for prolonged circulation time, controlled drug release, and synergistic chemo-photothermal therapy. The prepared NPs had a diameter of ~150 nm, core-shell structure, high drug load (~92 wt%), and decreased uptake by RES. Compared to individual treatment, the NPs under dual stimuli showed high increased apoptosis in tumor cells [65]. PAX-loaded biocompatible nanosystems derived from human ER membranes were constructed to enhance the targeting of the drug for the treatment of gastric cancer. With a lipid insertion method, a phospholipid derivative, 1,2-distearoyl-***sn***-glycero-3-phosphoethanolamine-*N*-(maleimide[polyethyleneglycol]-3400) (DSPE-PEG-MAL) was inserted into the ER membrane-derived tumor-targeting molecular vesicles. The spherical and uniformly sized (171.7 ± 4.7 nm) nanosystems were obtained which exhibited biphasic release pattern of the drug. When subjected to in vivo testing in Balb/c nude mice, the nanosystems showed accumulation of the drug into the tumor site within 2 h of the administration reducing the tumor volume to 61% [66]. In a similar study, folic acid (FA) as a tumor-targeting molecule and magnetic nanoparticles (MNP) were coated on the RBC-surface using lipophilic interaction and chemical conjugation techniques, respectively. MNPs were functionalized using DSPE-PEG-FA. Functionalized MNPs were conjugated with RBC ter 119 antigen. These engineered RBCs quickly adhered to the circulating tumor cells (CTC) and conjugated to form RBC-CTC conjugates which released the CTCs upon treatment with RBC-lysis buffer followed by centrifugation [67]. The targeted delivery of DOX in the treatment of liver cancer was also studied in another report. The surface modified NERs loaded with DOX were prepared and their surfaces were modified with FA and PEG for their tumor targeting ability. Compared to the conventional DOX injection, FA-PEG-DOX-Nano-RBCs were reported to prolong the drug circulation time in vivo and augmented the concentration and accumulation of drug in liver tumor cells [68].

A novel, targeted, biomimetic nanoplatform for combined cancer chemotherapy and phototherapy was achieved by embedding the BSA encapsulated with 1,2-diaminocyclohexane-platinum (II) and ICG for targeting peptide-modified erythrocyte membrane in order to improve the tumor internalization. The nano-therapeutic system had tumor-specific targeting ability with efficient removal of tumors, as well as suppression of metastasis in vivo by combined photothermal therapy and chemotherapy under phototriggering [69]. A nanoworm, biomimetic NER for siRNA delivery has also been reported in literature wherein, the RBC membrane cloaking protected siRNA from degradation by RNAse A. The siRNA vector stayed longer in the blood circulation than that of both negatively charged BSA spheres, as well as positively charged BSA and showed the ability to escape from late lysosomes and endosomes in order to achieve efficient transfection for knockdown of genes. These biomimetic worm-like NERs charge reversible gene vector is a novel method for much effective in vivo siRNA therapy [70].

The magnitude of research carried out on the delivery of drugs bound to erythrocytes emphasized more on controlled release, improved pharmacokinetics and pharmacodynamics profiles of drugs such as anti-neoplastics, anti-inflammatory, anti-thrombotics, and anti-microbials, etc. [71]. Poised with unique advantages in drug transportation, RBC-based systems were further developed and in yet another study, an albumin bound NIR dye and DOX as a chemotherapeutic agent were co-encapsulated inside the RBCs and their surfaces were modified using target protein to achieve tumor targeting. The RBC membranes were destroyed using an external NIR laser and light-induced photothermal heating which resulted into the release of drugs effectively. This work was novel, smart, and highly promising in the field of targeted combination therapy of cancer (photothermal and chemotherapy) [72]. Similarly, the immunocompatible nanocarriers cloaked in RBC membrane were utilized for drug targeting. DOX-loaded PLGA NPs were prepared using double emulsification method and RBC membrane vesicles were prepared using a sonication method. Biomimetic RBC-NPS were prepared by fusing DOX-loaded PLGA NPs into RBC membrane vesicles. The prepared biomimetic-NPs of DOX shrouded inside the RBC membranes showing good inhibition of tumor growth as compared to the treatment with free drug [73]. Recently, dual-functional exosome-based super-paramagnetic NP-clusters were developed for the cancer therapy and the in vivo studies on murine hepatoma-22 subcutaneous cancer cells demonstrated the suppression of tumor growth by these novel drug carriers under an external magnetic field [53].

The photothermal therapy (PTT) is a highly efficient method for the induction of tumor neoantigen release in situ and it has great potential to be used in the cancer immunotherapy. Nanovesicles of biomimetic black phosphorous quantum dots (BPQD) coated with ER membranes (RM) nanovesicles [BPQD-RMNV] exhibited longer circulation time and better tumor accumulation in vivo in breast cancer cells. The BPQD-RMNV mediated PTT combined with anti-program death-1 (aPD-1) treatment markedly deferred residual and metastatic cancer growth in vivo. Therefore, the combination of BPQD-RMNV-mediated PTT with immune checkpoint-blockade antibodies were found to improve the permeation and efficacy of CD8^+^ T cells inside the tumor restraining the growth of basal-like breast cancer cells in vitro [74]. In another study, graphene oxide NPs were prepared by incorporating ICG as photosensitizer along with DOX as the chemotherapeutic agent and the shell was made of RBC-membrane inserted with FA as targeting molecule. These NPs showed excellent biocompatibility and remarkable ability to evade the RES clearance [75]. Hybrid membranes of various kinds of cells could efficiently be coated onto the surface of NPs in order to achieve the desired functions [76]. The coating using cell membranes has emerged as an upcoming strategy for improving the properties of nanomaterials. In lieu of this, RBC membrane was fused with MCF-7 breast cancer cell membrane and hybrid membrane-camouflaged melanin NPs were prepared for improving the therapeutic potential of PTT. Melanin-RBCs showed both prolonged blood circulation time and homotypic targeting to the MCF-7 breast cancer cells [76].

### 4.5. NERs in Glioma Therapy

Amongst all cancers, glioma is the most fatal with limited options of treatment and a very low survival rate. It is extremely challenging for NPs to cross blood brain barrier (BBB) and blood brain tumor barrier (BBTB). Researchers recently designed RBC-membrane-coated solid-lipid-NPs-based nanocarrier system with T-7 and NGR peptide to counter these challenges, to evade the BBB and to reach the glioma. The NPs of peptides T-7 and NGR were prepared using DSPE-PEG and fused with RBC membrane. These biomimetic nanocarriers showed enough potential to deliver the model drug vincristine to the brain [77]. Similarly, RBC-membrane-coated-nanocrystals modified with the tumor-targeting peptide c(RGDyK) were developed for their potential to treat glioma. Docetaxel-loaded nanocrystals were prepared using streptavidin-PEG-DSPE and biotin-DSPE-c(RGDyK) and fused with RBC membrane. These nanocarriers showed improved tumor accumulation and enhanced therapeutic efficacy in vivo in mice with subcutaneous tumor and orthotropic glioma. These nanosystems displayed higher drug loading, excellent biocompatibility, long-term stability, and were found to be suitable for brain delivery [78]. RBC-hitchhiking is another valuable technological tool and a new formulation development strategy of complexing NPs with the RBCs for the treatment of aggressive and small cancers, including lung cancer [79].

### 4.6. NERs in Overcoming Drug Resistance

RBC-membrane-derived vesicles (RDV) coated poly (acrylic acid) cystamine hydrochloride-*D*-α-tocopherol succinate (PAAssVES) NPs were developed using emulsification method followed by solvent-volatilization method and finally was loaded with anticancer drug sorafenib (SFN). The obtained RDV-coated NPs (RDV-NP) showed excellent stability and particle size of 113.5 nm with −10.7 mV zeta potential. In comparison to the free SFN treatment, RDV-NPs demonstrated longer circulating time [80]. To overcome MDR in breast cancer treatment, NERs could be an ideal approach for the co-delivery of anticancer drugs with a p-gp inhibitor. In lieu of this, FA-modified NER system was developed to simultaneously transport tariquidar and paclitaxel and evaluated for its in vitro and in vivo properties. The nanocarrier was observed to be of size 159.8 ± 1.4 nm and zeta potential −10.98 mV, respectively. It showed a sustained release of paclitaxel within 120 h in both pH 6.5 and 7.4 media. Cytotoxicity studies on MCF-7 cells showed that both paclitaxel and tariquidar were able to inhibit the proliferation of MCF-7 cells and pharmacokinetic studies revealed prolonged drug release for all nanoformulations [81]. For the development of nanomedicine to target gliomas, it is important for them to have the capability to cross the BBB, as well as the BBTB. In this light, a preparation based on PLGA-NPs coated with ER-membrane with dual-modified peptide ligands was developed. Euphorbia factor-L1 was used as model drug, and these NPs were taken up by the cells and showed the ability to cross both BBB and BBTB, thereby producing cytotoxic effects in vitro. Furthermore, in vivo studies supported these results and the prepared NPs could enter the brain, targeting tumor tissues and significantly extending the life span [82]. Subsequently, NERs of 5-FU for liver cancer treatment were prepared for improved pharmacokinetic and biodistribution of 5-FU. The 5-FU-loaded chitosan coated–poly (lactide-co-glycolic acid)-NPs-NEs chitosomes had narrow particle size distribution, desirable encapsulation efficiency and sustained release profile of up to 72 h, which suggested that these nanosystems were able to deliver 5-FU with enhanced targetability to the liver cells [83]. In another recent study, the surface of NERs was decorated with two different flourophores: 7-amino-4-methylcumarin and dibenzocyclooctinecyanine with glutaraldehyde. The NER-derivatives exhibited higher stability in physiological conditions [84].

In vitro uptake by macrophages, in vivo biodistribution and pharmacokinetic studies demonstrated that the RBC-membrane is a better alternative to PEG, which is currently the gold standard for nanoparticles “stealth”. The combination of natural cell membranes with synthetic NPs poses a novel and biomimetic strategy for the designing of nanomaterials which additionally displays a wide range of biomedical applications, owing to the properties of functional materials used [85]. The biomimetic-NPs have attracted increasing attention in pharmaceutical and biomedical applications by imitating the structures and the functions of biological systems including longer blood circulation time. Nevertheless, the circulation time of these NPs is still far lesser than that of the native cells, which limits their applications greatly [86,87]. Accordingly, a camouflage comprising ER-membranes renders NPs as a platform that has combined advantages of native ER-membrane as well as of nanomaterials. When, they are injected into the animal models in vivo, the ER-membrane-coated NPs imitate and behave like RBCs and interact with the surrounding macromolecules to achieve longer circulation time [88].

## 5. Applications of NERs in Non-Cancer Therapies

NERs have been extensively investigated for the therapy and diagnosis of various forms of cancers and that has further encouraged the researchers to test their applications for non-cancer diseases. The findings are interestingly very promising which has opened a new vista for the applications of NERs. In one such study, the NERs of antimalarial drug pyrimethamine (PRY) were developed by sonication method which showed good stability and controlled in vivo release. Developed NERs improved the treatment of malaria when combined with artemisinin drug [89]. In another interesting research, artesunate (ART) conjugated NERs were prepared to increase the stability, decrease the toxicity, cost, and drug leakage for the treatment of malaria. The optimized ART-NER nanoformulations were non-aggregated, uniformly sized, with drug loading of 25.20 ± 1.3 µg/mL and when administered IV, showed higher plasma drug concentration as compared to free drug in vivo [90]. For the treatment of atherosclerosis, the nanomaterials can be explored and it is believed that they can serve as a powerful tool in its treatment. In an attempt to achieve this, biomimetic, well defined core-shell-nanocomplexes of rapamycin (RAP)-loaded PLGA-NPs cloaked with RBC were created with negative surface charge. The prepared nanosystems were shown to be safe and effective in the management of atherosclerosis as the biomimetic behavior of RBCs resulted in decreased macrophage-mediated phagocytosis and increased aggregation of NPs in the atherosclerotic plagues using targeted delivery [91]. Most of the applications of NERs were limited to the IV, IP, and sub-cutaneous (SC) routes of administration. To explore other possible routes of administration, they were also investigated as inhalational drug carriers in few studies successfully. In one such study, NERs were conjugated with CARSKNKDC (CAR), a cell permeable peptide and fasudil, a rho-kinase (ROCK) inhibitor using hypnotic-lysis and extrusion method for prolonged pulmonary vasodilation. The NPs obtained were of spherical shape with average size 161.3 ± 1.37 nm and % entrapment efficiency (EE) 48.81 ± 1.96%, which showed stability of around 3 weeks at 4 °C, and the drug fasudil was shown to be released in a controlled release pattern for more than 48 h. The reduction in the pulmonary arterial pressure upon intratracheal administration of CAR-fasudil-NERs was approximately 2-fold more specific to the lungs in comparison to fasudil alone [92]. Few other studies in the literature also suggest that the NERs can effectively be utilized for inhalational delivery of fasudil drug as cell-derived carriers [93,94].

## 6. Biosensing Applications of RBC-Mediated Carriers Systems

The cutting-edge RBC-mediated anti-cancer drug delivery by NERs is well established along with their applications in some of the non-cancer diseases. Additionally, the in vivo nanobiosensor-based theragnostic application of RBCs has emerged as a topic of interest in present days for advanced medical diagnostics, analytical chemistry, and environmental monitoring [95,96,97]. Their extraordinary profusion, mobility, and loading capacity makes them an attractive tool for sensing the analytes present in the blood. The sensor-loaded ERs or the dubbed erythrosensors can be re-infused in the blood and can be used for the measurement of analyte levels in the blood stream [96]. Previously, 3-D focusing of RBCs in microchannel flows for bio-sensing applications has also been reported [98]. Additionally, the fluorescent sensors were incorporated inside ERs followed by non-invasive monitoring to follow changes in plasma analyte concentrations [99]. For the management of diseases such as diabetes, there exists a need for long-term, minimal-invasive system to monitor blood glucose as analyte. Currently employed methods suffer from disadvantages of low patient compliance for the finger stick test and require regular calibrations for continuous glucose monitoring. The RBCs can act as a biocompatible carrier of sensing assays for such long-term monitoring procedures. They can serve as long-term (˃1 to 2 months), continuously circulating biosensors in the diagnosis and management of such long term diseases [100]. Similarly, the fusion of quantum dots (QD) with living cell membranes for bio-sensing capability in imaging technique is a flexible approach for controlled, hydrophobic QDs-based fluorescence analysis of living cellular membranes [101].

## 7. Recent Patents on NERs for Cancer Therapy

In this section, the important and recent patents related to the use of NERs as bioactive carriers in cancer therapy are summarized. The description of the invention and its pharmaceutical advantages are mentioned in Table 2 for the quick understanding of the readers. The recent patents enlisted here show the viability of this approach for delivering the antineoplastic drugs to their required targeted site of action. The enhanced potential of the antineoplastic drugs loaded in NERs carrier system points towards the growth of this novel drug delivery system in cancer therapy [102,103,104,105,106,107,108,109,110,111].

## 8. Recent Clinical Trials on Anticancer Drug-Loaded NERs

The comprehensive and detailed investigations on the RBC-mediated drug delivery strategy have grown rapidly and, currently, the research and development on these strategies have transformed and advanced to the pre-clinical and clinical phases. None of the anti-cancer drug loaded-RBC-technology has reached the market till now, however, few clinical trials on RBC-based technology have been conducted for dexamethasone-21-phosphate in ataxia telangiectasia, asparaginase in pancreatic cancer, lymphoblastic leukemia, thymidine phosphorylase in mitochondrial neuro-gastrointestinal encephalomyopathy and RTX-134 in phenylketonuria, etc., as reported [112]. A phase IIb open-label study on ER-encapsulated asparaginase (Eryaspase) in combination with chemotherapy in second-line advanced pancreatic adenocarcinoma [NCT02195180] is being carried out [113]. Eryaspase in combination with chemotherapy (gemcitabine) was associated with improvements in overall survival (OS) and progression-free survival (PFS), irrespective of asparagine synthetase (ASNS) expression in second-line advanced pancreatic adenocarcinoma. A Phase III trial is underway [114]. An open-label, randomized, multicentered, phase-III trial on Eryaspase in patients with pancreatic ductal adenocarcinoma and those who failed only one prior-line of systemic anticancer therapy for advanced stage of pancreatic cancer were also reported [NCT03665441] [115]. Another clinical trial on Eryaspase was a randomized phase II/III investigation by combining carboplatin and gemcitabine chemotherapy versus chemotherapy-alone as first-line treatment in metastatic or locally recurrent triple-negative breast cancer [NCT03674242] patients [116]. A clinical trial study on PEGylated-asparaginase-encapsulated in erythrocytes for patients with acute lymphoblastic leukemia (ALL) is also underway [NCT03267030] [117] as shown in Table 3.

## 9. Conclusions

The NERs are the RBC-derived nanocarrier systems having extraordinary features among which the long blood circulation time, controlled release of drug over a long period of time, ability of escaping the immune system and targeted delivery of drugs to the site of action are the main features. These properties led to an extensive investigation on NERs and the results have shown promising potential for NERs to be developed as a novel, biomimetic and efficient drug delivery system for the effective and targeted delivery of drugs including anticancer drugs and others. In addition, NERs have also been studied as a powerful diagnostic tool for the diagnosis of various tumors. The encouraging findings of the in vitro and in vivo studies conducted on NERs further lead to the advancement to clinical trials stage and the efficacy and safety is being tested on human beings. However, the NERs still suffer from the disadvantages related to scalability and validation which is one of the limiting factors for its industrialization. Nevertheless, many studies are underway to utilize the NERs as a multifaceted drug delivery tool and it has shown substantial capabilities of being developed and applied for human use.

## Figures and Tables

**Table 1:** Anticancer nanoformulations of nanoerythrocytes (NER), their surface modification and specific tumor targeting ability.

Name of The Anticancer Drug/Agents	Type of RBC-Based Nanoformulation	Type of Surface Modification/Functionalization/Ligands	Reported Applications	Ref.
DAU	NERs	DAU was covalently linked by glutraldehyde to the NERs	CDF1 leukemia tumor	[32]
DOX	RBC-Iron oxide NPs	Pre-coated with chlorine^6^	For imaging-guided combined photodynamic and chemotherapy of cancers	[42]
ICG and Perfluorocarbon (PFC)	RBC membrane cloaked albumin NPs	--	Ideal for clinical cancer phototherapy treatment	[43]
Sodium Transhione II A sulphonate	Drug loaded nano-RBCs	--	Nanosystem was better than conventional injection in-vivo	[44]
Campothecin	RBC-membrane loaded nanovesicles	Labelled non-covalently with amphiphilic fluorophore	--	[46]
5-FU	Biomimetic nanoerythrocyte- membrane–chaperoned liposomes	--	Hepatocellular carcinoma	[47]
Engineered *E. coli* sp.	Biohybrid microswimmers (RBC-NERs)	Conjugation of streptavidin-modified bacteria with biotin-modified-NERs using non-covalent streptavidin interaction	Targeted cargo delivery system	[50]
Antigenic peptide Hgp 10025-33	Erythrocyte membrane enveloped PLGA-NPs	Mannose-inserted membrane structure was constructed to actively target antigen presenting cells in lymphatic organs	Cancer nanovaccine in cancer immunotherapy	[51]
Co-delivery of PAX and DOX	Magnetic *O*-Carboxy methyl chitosan NPs	Camouflaged with an Arg-Gly-Asp anchored ER-membrane	Better tumor uptake	[52]
PAX	Biomimetic polymeric NPs	--	4T1- breast cancer cell membrane	[54]
Curcumin (CUR)	NERs	--	Enhanced antitumor activity	[56]
Gambogic acid	Biomimetic RBC-membrane coated PLGA NPs	--	Colorectal cancer	[58]
FA	Upconversion NP coated with RBC-membranes	Surface-modified with ligands for active targeting of cancer cells	For in-vivo cancer imaging	[60]
				
DOX	Drug loaded RBC-membrane coated copper sulphide NPs	--	100% melanoma tumor growth inhibition rate	[64]
10-Hydroxy Campothecin and ICG	Biomimetic RBC membrane nanovesicles	--	Synergistic chemo-photothermal therapy	[65]
PAX	Encapsulated in human erythrocyte membrane	A phospholipid derivative was used for tumor targeting into ER-membrane derived nanovesicles	Gastric cancer	[66]
FA	Magnetic NPs coated on the surface of RBCs	Chemical conjugation and hydrophobic interactions between RBC-circulating tumor cells	Enhanced tumor targeting ability	[67]
DOX	NERs	Surface modified by FA and PEG	Enhanced tumor targeting ability in vivo in liver cancer	[68]
siRNA	Nanoworms, biomimetic NERs	-	Efficient siRNA therapy in vivo	[70]
DOX	Coencapsulated inside RBCs	Albumin bound NIR dye	Combinational photothermal and chemotherapy of cancer	[72]
DOX	RBC-cloaked membrane	--	For the treatment of solid tumors	[73]
DOX	NPs of graphene oxide-DOX-RBC-membrane- ICG as photosensitizer	FA	Had excellent ability to evade RES	[75]
Vincristine	RBC-membrane coated solid lipid NPs	T7 and NGR peptide	Brain delivery for treatment of gliomas	[78]
Codelivery of PAX and Tariquidar	Nano-erythrocytes (NEs)	FA modified NEs	Breast cancer management	[81]
Euphorbia factor L1	PLGA-NPs coated with ER-membrane	Dual–modified peptide ligands	Brain delivery for gliomas	[82]
5-FU	FU –loaded chitosan-coated-PLGA –NPs-NE-chitosomes	--	Liver targeting	[83]

**Table 2:** Recent patents available on NERs as carrier system for cancer therapy.

Patent Number	Invention Title	Description of The Invention	Pharmaceutical Advantages	Ref.
US5653999	NERs as bioactive carrier	A complex comprising of a bioactive agent coupled to vesicles derived from ERs. Prepared ERs had size less than 1 µm and substantially free of hemoglobin.	DAU–NERs conjugate had a higher antineoplastic activity than the free bioactive agent.	[102]
WO1998011919A3	Polyethylene glycol conjugated NERs, method of making same and use thereof.	Long circulating NERs avoid rapid clearance by RES.	Prolonged circulation time.	[103]
US20040180094A1	Activation agents on the surface of encapsulation vesicles	Target ligands can be synthetic, semi-synthetic and naturally occurring such as antibiotics, hormones, lectins, glycoproteins, peptides, amino acids, polypeptides, sugars, saccharides, carbohydrates, cofactors, bioactive agents, and genetic materials such as nucleotides and nucleosides, etc.	The present invention addressed drug resistance problems in vivo by attaching optimal target ligand to encapsulation vesicles.	[104]
US8211656	Biological targeting composition and methods of using the same.	Targeted delivery of imaging agents, drugs, peptides, proteins, and pharmaceuticals using modified RBCs is described here.	Modified RBCs can carry a variety of therapeutic moieties for the treatment of various ailments including cancer.	[105]
US20130202625	Use of human erythrocytes for prevention and treatment of cancer dissemination and growth.	Cancer metastasis specially breast cancer metastasis can be prevented by blocking the circulation of metastatic cells and by blocking angiogenesis such as capturing endothelial progenitors that are recruited to the tumor, or by physically blocking of the capillaries of the tumor or the metastasis.	RBCs have potential for use as therapeutics as they are easily retrieved from a patient, non-immunogenic, and are biologically designed to navigate the microcirculation including tortuous tumor vasculature.	[106]
US20200138987	Composition for material delivery including exosome mimetics derived from RBCs, and use thereof.	Exosome mimetics derived from RBCs are used for material delivery such as drug, radioactive material and fluorescent materials, etc.	Exosomes (small vesicles, 30nm-100nm) have drawn attention as new drug delivery carrier system for targeted delivery to a specific organ and can be used as imaging tools.	[107]
US20200289666	Biomimetic anisotropic polymeric particles with naturally derived cell membranes for enhanced drug delivery.	Biomimetic particles can be used in the treatment of excessive bleeding, thalassemia, thrombopenia, cancer, infectious diseases, etc.	Particles comprised of polymeric core of defined shape, size, surface from naturally derived cell membranes such as RBCs, have application in drug delivery and cell engineering.	[108]
US20170367990	Use of NPs coated with RBC membranes to enable blood transfusion.	An inner core of nanoparticle comprised of non-cellular compound and an outer surface comprise of cellular membrane derived from RBCs.	Suitable in blood transfusion for giving a blood-source with a mismatched type of blood, or potentially a mismatched blood type to a recipient.	[109]
US10596197	Red blood cell membrane derived microparticles and their use for the treatment of lung diseases.	Treatment with RBCs-MPs to the lung through inhalational route promoted the expression of immune regulatory cytokines including IL-10 and reduced inflammatory responses and injury to the lungs.	Have remarkable potential as immuno-modulating agent for a number of lung disorders such as chronic-obstructive pulmonary disorder (COPD), bronchitis, acute lung injury, pulmonary fibrosis, etc.	[110]
US20170095510	Use of NPs coated with red blood cell membranes to treat hemolytic diseases and disorders.	Hemolytic diseases are auto-immune disorders caused by an attack of said mammal RBCs by said mammal’s own body or in between pregnant mammal and fetus RBCs.	Invention will be employed in nano-engineering, molecular biology, etc.	[111]

**Table 3:** List of clinical trials available for asparaginase encapsulated in erythrocytes (Eryaspase).

Clinicaltrial.gov Identifier	NCT03674242	NCT03665441	NCT02195180	NCT03267030
Drug encapsulated in erythrocyte	Asparaginase encapsulated in erythrocytes (Eryaspase)			
Eryaspase combined with other anti-cancer drugs	Eryaspase combined with gemcitabine or carboplatin	Eryaspase combined with either gemcitabine plus abraxane, or irinotecan-based therapy	Eryaspase combined with gemcitabine or 5-fluoro-uracil/oxaliplatin/leucovorin (FOLFOX)	Eryaspase combined with GRASPA
Purpose	Treatment	Treatment	Treatment	Treatment
Cancer type	Triple negative breast cancer	Pancreatic adenocarcinoma	Progressive metastatic pancreatic carcinoma	Acute lymphoblastic leukemia
Recruitment status	Recruiting	Active, not recruiting	Completed	Completed
Sponsor	ERYtech Pharma	ERYtech Pharma	ERYtech Pharma	Birgitte Klug Albertsen
Study-type	Interventional	Interventional	Interventional	Interventional
No of participants	64	500	141	55
Allocation	Randomized	Randomized	Randomized	N/A
Intervention model	Parallel assignment	Parallel assignment	Parallel assignment	Single group assignment
Masking	Open label	Open label	Open label	Open label
Phase	Phase 2/3	Phase 3	Phase 2	Phase 2
Start of the study	June 2019	September 2018	July 2014	August 2017
Completion of the study	October 2020	October 2021	November 2017	October 2020

## Data Availability

This study did not report any data.
